# A study on predictive nomogram for abdominal wall hernia in peritoneal dialysis patients based on multicenter data

**DOI:** 10.3389/fmed.2025.1624861

**Published:** 2025-12-09

**Authors:** Yugang Cao, Tao Fang, Jun Guo, Sha-sha Peng, Jin Yang

**Affiliations:** Hubei Key Laboratory for Kidney Disease Pathogenesis and Intervention, Department of Hepatobiliary Surgery, Huangshi Central Hospital, Hubei Polytechnic University School of Medicine, Huangshi, Hubei, China

**Keywords:** peritoneal dialysis, end-stage renal disease, abdominal wall hernia incidence, prediction, nomogram

## Abstract

**Purpose:**

This study aims to develop and externally validate a scoring nomogram based on three key indicators (Peritoneal dialysis vintage, age, and albumin) to predict the risk of developing abdominal wall hernia in patients with end-stage renal disease (ESRD) undergoing peritoneal dialysis.

**Methods:**

A total of 480 patients undergoing peritoneal dialysis from three medical centers were enrolled in this study and randomly assigned into a training cohort (*n* = 300) and an external validation cohort (*n* = 180). To identify independent risk factors associated with the development of abdominal wall hernia in patients with end-stage renal disease receiving peritoneal dialysis, univariate and multivariate logistic regression analyses were performed using IBM SPSS Statistics version 26.0 (IBM Corp., Armonk, NY, USA) and R software version 4.2.1 (R Foundation for Statistical Computing, Vienna, Austria). A predictive nomogram was subsequently developed based on significant predictors identified through multivariable logistic proportional hazards regression modeling. The model’s predictive performance was externally validated and comprehensively evaluated using the area under the receiver operating characteristic curve (AUC), calibration plots, decision curve analysis (DCA), and Kaplan-Meier survival analysis.

**Results:**

Gender, BMI, hemoglobin, ultrafiltration volume, diabetes, serum creatinine, and catheter insertion method showed no significant association with hernia development. PD vintage (*P* < 0.01), PD modality (*P* < 0.01), serum albumin (*P* < 0.01), and age (*P* < 0.01) were significantly associated. Multivariate analysis confirmed PD vintage (OR = 11.09; 95% CI: 3.64–63.59), albumin (OR = 0.24; 95% CI: 0.11–0.41), and age (OR = 1.20; 95% CI: 1.15–1.27) as independent predictors. The nomogram achieved an AUC of 0.823 in the training cohort and 0.747 in the validation cohort. Risk stratification showed significant differences in hernia-free survival between low- and high-risk groups (*P* < 0.001).

**Conclusion:**

This study successfully developed and externally validated a novel nomogram with high accuracy that can effectively predict the risk of abdominal wall hernia in patients with end-stage renal disease undergoing peritoneal dialysis.

## 1 Introduction

Peritoneal dialysis (PD) has become a widely adopted renal replacement therapy for patients with end-stage renal disease (ESRD), valued for its ability to preserve residual kidney function, offer flexible treatment modalities, and support home-based care. However, as PD duration and global prevalence rise, PD-related complications have emerged as critical factors affecting patient outcomes. Among these, abdominal wall hernia stands out as a frequent and clinically significant mechanical complication, with reported incidence rates ranging from 10% to 25% and up to 30% in long-term PD populations (1–3). Epidemiological studies indicate that patients who develop abdominal wall hernia face a twofold increase in hospitalization risk and a 1.5-fold higher mortality rate compared to those without herniation. The presence of hernia not only causes physical discomfort and functional decline but also disrupts dialysis efficiency, potentially leading to life-threatening complications such as bowel incarceration, intestinal obstruction, and necrosis-often necessitating surgical intervention. Despite growing awareness, current risk stratification tools remain insufficiently precise for individualized prediction. Therefore, there is a pressing clinical need for a validated, accurate predictive model to identify high-risk patients early and guide preventive strategies. This study addresses that gap by developing and externally validating a novel nomogram specifically tailored to predict abdominal wall hernia risk in PD patients, with the potential to improve clinical decision-making and patient outcomes.

Numerous studies have identified potential risk factors associated with the occurrence of abdominal wall hernia in PD patients, including prolonged duration of PD, advanced age, hypoalbuminemia, obesity, and specific dialysis modalities such as continuous ambulatory peritoneal dialysis (CAPD) (4–6). Despite these findings, the current understanding remains fragmented, and existing models for predicting the risk of abdominal wall hernia in this population are limited by small sample sizes, single-center designs, and a lack of external validation. Moreover, most of these models lack clinical applicability due to insufficient discrimination and calibration performance, which hampers their utility in guiding individualized risk assessment and preventive strategies.

In this context, the development of a robust, externally validated, and clinically applicable predictive tool is urgently needed to facilitate early identification of high-risk patients and to support targeted preventive interventions. Nomograms, as graphical predictive models that integrate multiple prognostic factors, have demonstrated considerable value in various clinical settings by enabling individualized risk stratification and improving clinical decision-making. The present study aims to develop and externally validate a nomogram for predicting the risk of abdominal wall hernia in PD patients based on multicenter retrospective data. By identifying independent predictors and rigorously validating the model’s performance, this tool holds promise for enhancing risk stratification, guiding timely interventions, and ultimately improving clinical outcomes in this vulnerable patient population.

## 2 Materials and methods

### 2.1 Study subjects

This study employed a retrospective approach to screen patients. The training cohort data were obtained from patients with ESRD treated at Huangshi Central Hospital between October 2014 and October 2024. The external validation cohort data were collected from patients treated at Huangshi Chinese Medicine Hospital and Huangshi Central Hospital Puai Campus during the same period. The research team conducted a retrospective review of medical records of patients diagnosed with renal insufficiency who underwent peritoneal dialysis during their hospitalization. All cases of concomitant abdominal wall hernias were confirmed via imaging modalities, including computed tomography (CT), magnetic resonance imaging (MRI), or B-mode ultrasound.

To ensure a homogeneous study population, the following inclusion criteria were strictly applied: (1) age between 14 and 80 years; (2) no prior history of abdominal surgery other than peritoneal dialysis catheter placement; and (3) no previous diagnosis of abdominal wall hernia or intra-abdominal hernia before initiation of peritoneal dialysis.

Exclusion criteria were established to minimize confounding factors and included: (1) occurrence of hernia unrelated to the peritoneal dialysis period; (2) presence of connective tissue disorders, cirrhosis with ascites, or long-term corticosteroid therapy; (3) prior major abdominal surgery or previous hernia repair; (4) follow-up duration less than 6 months or incomplete medical records with missing key variables; and (5) concurrent chronic cough, pregnancy, or severe cardiopulmonary disease that could influence intra-abdominal pressure.

**FIGURE 1:**
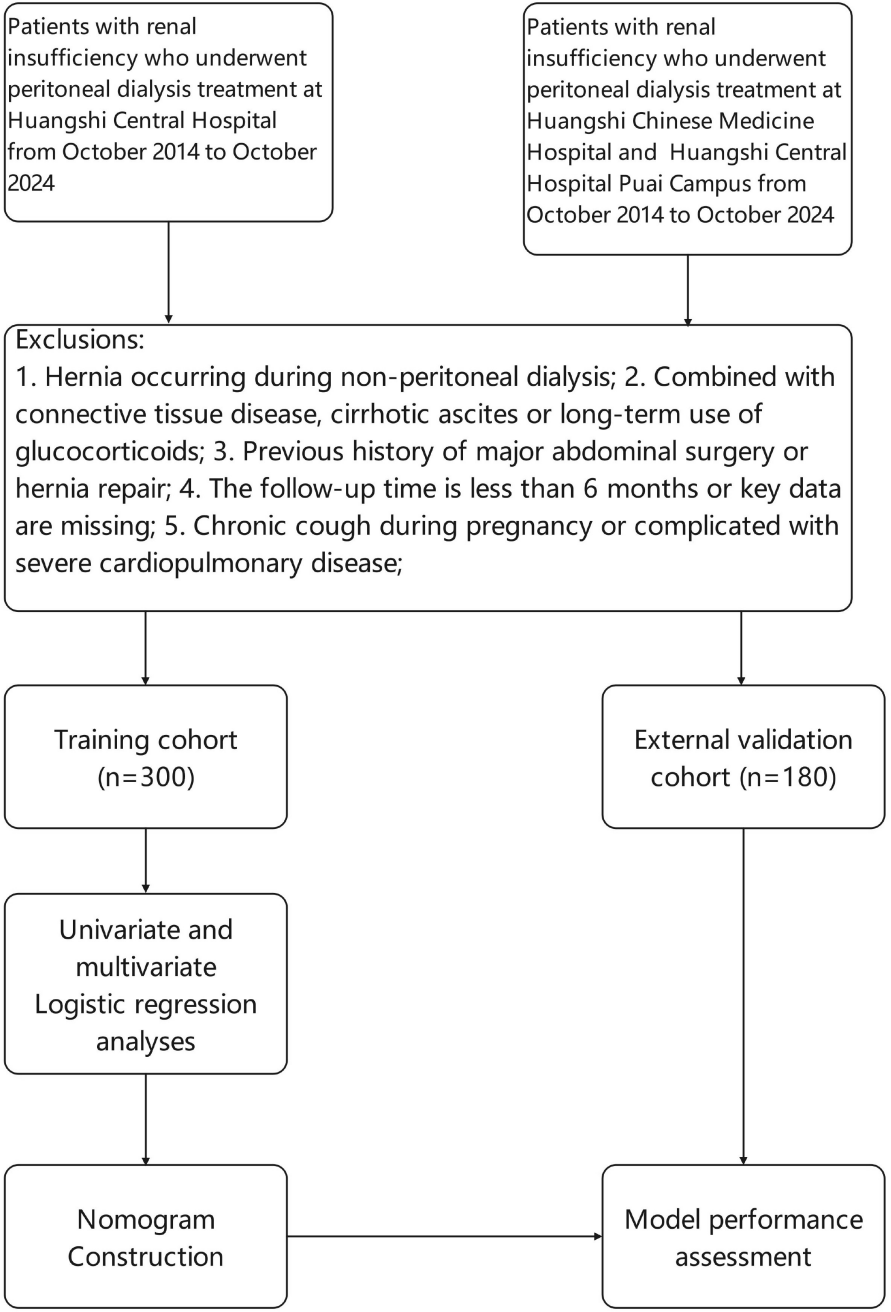
Flowchart showing the patient selection process, construction, and external validation of the nomogram.

After applying the aforementioned criteria, a total of 480 patients with renal insufficiency were enrolled in the study. Among them, 300 patients formed the training cohort, while the remaining 180 constituted the external validation cohort. The patient selection process is summarized in Figure 1, which outlines the flow diagram for cohort construction and external validation.

### 2.2 Data collection

Clinical and laboratory data from all patients with end-stage renal disease included in this study were retrospectively collected through medical record review. The variables collected included age, sex, creatinine level, body mass index (BMI), Peritoneal dialysis vintage (PDV), hemoglobin level, peritoneal dialysis catheter placement technique, ultrafiltration volume, type of abdominal wall hernia, peritoneal dialysis modality, serum albumin concentration, and hemoglobin concentration. All laboratory results were obtained within 1 week prior to surgery.

### 2.3 Follow-up

Post-discharge follow-up was conducted according to a predefined schedule: the first visit occurred 1 month after discharge, followed by bi-monthly visits during the first postoperative year and tri-monthly visits thereafter. Median follow-up was 25 months. Follow-up evaluations included measurements of serum creatinine, and albumin levels, as well as abdominal computed tomography (CT) scans. The time to hernia-free peritoneal dialysis was defined as the interval from the date of surgery to the detection of an abdominal wall hernia, whether identified via CT imaging or through clinical symptoms. All diagnosed abdominal wall hernias were confirmed using CT scans.

### 2.4 Statistical analysis

Continuous variables were summarized using means and standard deviations, while categorical variables were expressed as frequencies and percentages. To identify independent risk factors associated with hernia-free peritoneal dialysis, multivariable logistic regression analysis was performed, adjusting for potential confounders. A predictive nomogram was subsequently developed based on the independent predictors identified from the logistic regression model. The discriminative ability of the model in predicting hernia-free survival time was quantified using the area under the receiver operating characteristic (ROC) curve (AUC), which reflects the model’s capacity to distinguish between patients with and without the outcome of interest. Internal validation of the model’s calibration-assessing the agreement between observed outcomes and predicted probabilities-was conducted using calibration curves plotted against an ideal reference line. Furthermore, decision curve analysis (DCA) was employed to evaluate the clinical utility of the nomogram by estimating the net benefit across a range of probability thresholds, thereby providing insight into its practical applicability in clinical decision-making.

To facilitate risk stratification, X-tile software (version 3.6.1; Yale University, New Haven, CT, USA) was used to determine optimal cutoff values for dividing patients into low- and high-risk groups based on their nomogram derived risk scores. This approach was selected due to its robust algorithm that identifies the most statistically significant threshold while minimizing bias from arbitrary cutoff selection, thus enhancing reproducibility across studies.

Survival analyses for hernia-free peritoneal dialysis were conducted using Kaplan-Meier estimates, and differences between risk groups were evaluated using the Log-rank test. Univariate and multivariate logistic regression models were implemented in SPSS version 26.0 (IBM Corp., Armonk, NY, USA), while all graphical outputs (including ROC curves, calibration plots, DCA curves, and Kaplan-Meier survival curves) were generated using R statistical software version 4.2.1 (R Foundation for Statistical Computing, Vienna, Austria). Statistical significance was defined as a two-sided *P*-value < 0.05.

## 3 Results

### 3.1 Baseline characteristics

A total of 480 patients with end-stage renal disease undergoing peritoneal dialysis were enrolled in this study based on predefined inclusion and exclusion criteria. Of these, 300 patients constituted the training cohort and 180 patients formed the external validation cohort. All participants received peritoneal dialysis treatment. During follow-up, abdominal wall hernias developed in 51 patients (17%) in the training cohort and 25 patients (14%) in the external validation cohort. Among those in the training cohort, 18 cases (35.3%) were umbilical hernias, 26 (51.0%) were inguinal hernias, and 7 (13.7%) were incisional hernias. In the external validation cohort, the corresponding numbers were 7 (28.0%) umbilical hernias, 12 (48.0%) inguinal hernias, and 6 (24.0%) incisional hernias. The training cohort included 137 males and 163 females, whereas the external validation cohort consisted of 103 males and 77 females. The mean ages were 56 years in the training cohort and 59 years in the external validation cohort. Table 1 summarizes the demographic and clinical characteristics of patients undergoing peritoneal dialysis for end-stage renal disease in both cohorts.

**TABLE 1:** Baseline characteristics of PD patients.

Variables	Training cohort (*n* = 300)	External validation cohort (*n* = 180)
**Gender**		
Male	137 (45.7%)	103 (57.2%)
Female	163 (54.3%)	77 (42.8%)
Age (years)	56.00 ± 12.21	59.00 ± 11.63
BMI (Kg/m^2^)	22.37 ± 2.99	21.29 ± 2.43
PDV (years)	2.25 ± 1.10	2.15 ± 1.23
Creatinine (μmol/L)	545.49 ± 147.21	593.58 ± 128.38
**Diabetes**		
Yes	103 (34.3%)	54 (30%)
No	197 (65.7%)	126 (70%)
Albumin (g/L)	34.34 ± 1.94	33.54 ± 1.87
Hemoglobin (g/L)	9.45 ± 1.52	9.23 ± 1.56
**PDCM**		
LPDC	181 (60.3%)	97 (53.9%)
OPDC	119 (39.7%)	83 (46.1%)
**PDM**		
CAPD	265 (88.3%)	156 (86.7%)
APD	35 (11.7%)	24 (13.3%)
DUV (ml)	1058.48 ± 277.65	987.23 ± 144.58
**Type of hernia**		
Umbilical hernia	18 (35.3%)	7 (28%)
Inguinal hernia	26 (51%)	12 (48%)
Incisional hernia	7 (13.7%)	6 (24%)

Data are presented as *n* (%) or mean ± standard deviation.

### 3.2 Risk factor screening

Univariate logistic regression analysis revealed no significant associations between the occurrence of abdominal wall hernia and variables including gender, body mass index (BMI), hemoglobin level, ultrafiltration volume, diabetes mellitus, creatinine level, or peritoneal dialysis catheter insertion method in patients undergoing peritoneal dialysis (all *P* > 0.05; see Table 2). In contrast, PDV (*P* < 0.01), PDM (*P* < 0.01), serum albumin level (*P* < 0.01), and age (*P* < 0.01) were found to be significantly associated with the development of abdominal wall hernia during peritoneal dialysis.

**TABLE 2:** Univariate and multivariate logistic regression analysis of independent risk factors associated with disease-free survival (DFS) in the training cohort.

Variables	Univariate analysis		Multivariate analysis	
	*P*	OR (95% CI)	*P*	OR (95% CI)
PDV (years)	<0.01	5.74 (3.60∼10.11)	<0.01	11.09 (3.64∼63.59)
**Gender**				
Female	Reference			
Male	0.93	0.97 (0.53∼1.78)	0.71	0.77 (0.18∼3.02)
BMI (kg/m^2^)	0.17	1.07 (0.97∼1.18)	0.33	1.10 (0.90∼1.34)
Age (years)	<0.01	1.20 (1.15∼1.27)	<0.01	1.11 (1.04∼1.21)
**PDM**				
APD	Reference			
CAPD	<0.01	4.04 (1.69∼12.01)	0.92	0.92 (0.18∼5.79)
Hemoglobin (g/L)	0.85	0.98 (0.79∼1.21)	0.64	1.12 (0.69∼1.79)
Albumin (g/L)	<0.01	0.21 (0.12∼0.31)	<0.01	0.24 (0.11∼0.41)
DUV (ml)	0.50	1.13 (0.55∼2.24)	0.72	1.19 (0.46∼3.03)
**Diabetes**				
No	Reference			
Yes	0.76	0.91 (0.50∼1.67)	0.78	0.89 (0.40∼1.98)
Creatinine (μmol/L)	0.42	1.45 (0.62∼3.81)	0.26	1.91 (0.63∼6.29)
**PDCM**				
LPDC	Reference			
OPDC	0.92	0.97 (0.53∼1.77)	0.97	1.02 (0.46∼2.25)

Multivariate logistic regression analysis confirmed that PDV [odds ratio (OR) = 11.09; 95% confidence interval (CI): 3.64–63.59; *P* < 0.01], serum albumin level (OR = 0.24; 95% CI: 0.11–0.41; *P* < 0.01), and age (OR = 1.11; 95% CI: 1.04–1.21; *P* < 0.01) were independent risk factors for the development of abdominal wall hernia in this patient population (Table 2). The multivariate correlation analysis of all candidate variables related to abdominal wall hernia in peritoneal dialysis patients is visualized in a bubble diagram, which is provided in the supplementary material (Supplementary Figure 1) to further illustrate the correlation intensity and direction between variables.

### 3.3 Development and validation of a novel nomogram predicting the risk of abdominal wall hernia in peritoneal dialysis patients with end-stage renal disease

**FIGURE 2:**
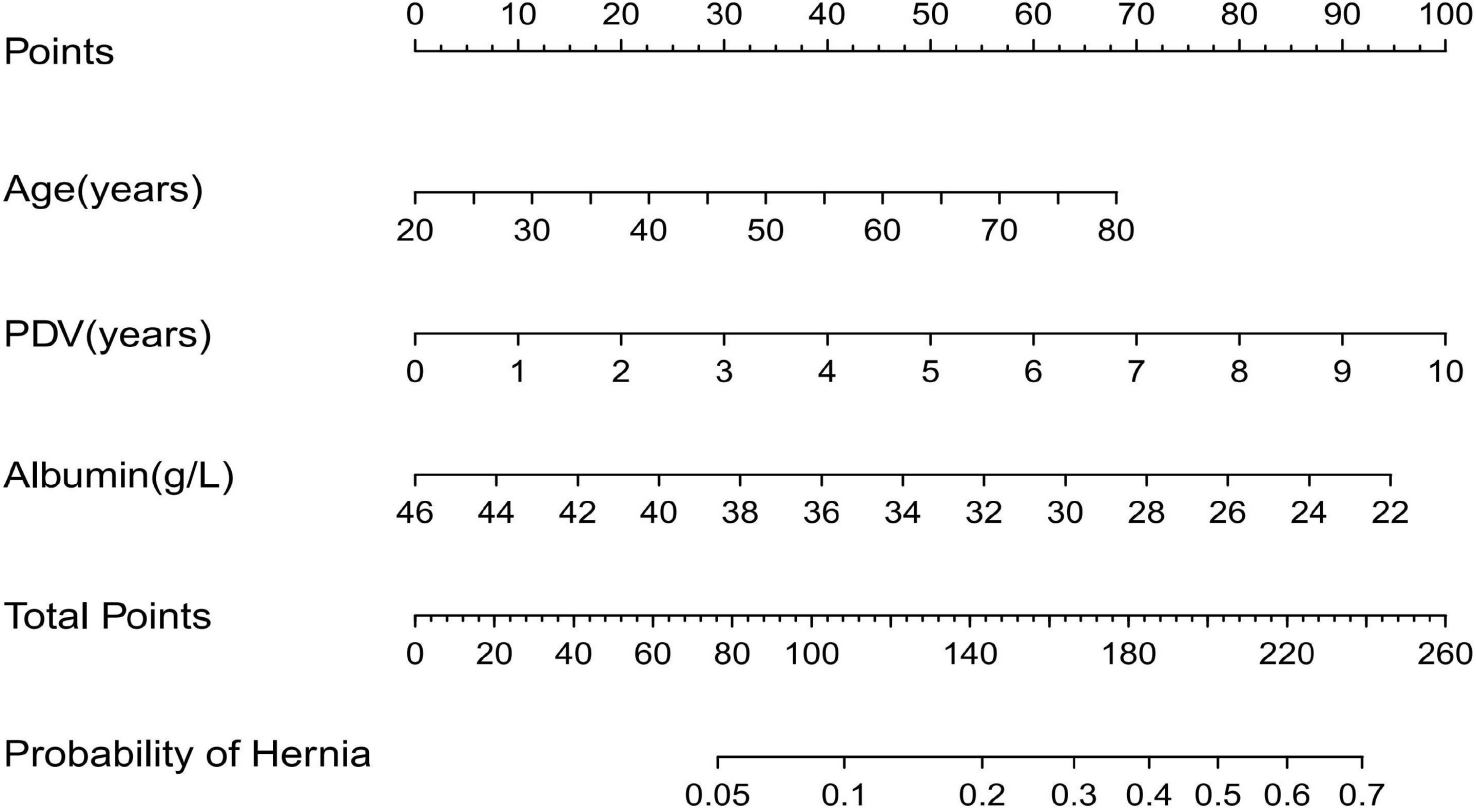
Nomogram to predict the incidence of abdominal wall hernia in PD patients.

**FIGURE 3:**
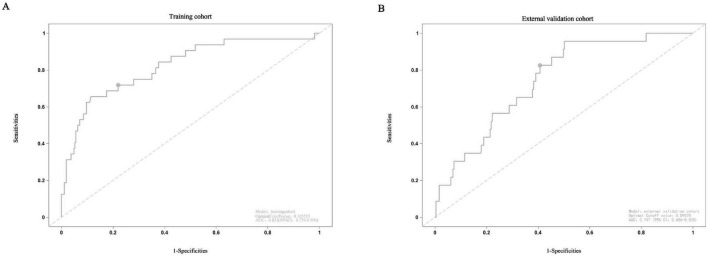
(A) Receiver operating characteristic (ROC) curves used to predict the occurrence of abdominal wall hernia in PD patients within training cohort. (B) ROC curves predicting the occurrence of abdominal wall hernia in PD patients within external validation cohort.

Based on the independent risk factors identified by multivariate logistic regression analysis, we developed a nomogram (Figure 2) to predict the probability of abdominal wall hernia in end-stage renal disease patients undergoing peritoneal dialysis. Each variable in the nomogram was assigned a points based on its regression coefficient, and the total points was calculated by summing individual points. The total points was then converted into a predicted probability of hernia occurrence using the nomogram’s calibration scale. The model demonstrated strong discriminative ability. As shown in Figure 3A, the area under the ROC curve (AUC) was 0.823(95% CI: 0.739–0.906) in the training cohort, indicating strong predictive accuracy. In the external validation cohort, the AUC remained satisfactory at 0.747 (95% CI: 0.656–0.838) (Figure 3B). These AUC values are either comparable to or surpass those of previously reported models for predicting hernia in peritoneal dialysis populations. For example, Zhang et al. developed a clinical risk score model based on body mass index and dialysis duration, which achieved an AUC of 0.715 (7), while Wang et al. applied a radiomics-based approach and obtained an AUC of 0.789 in a retrospective study (7).

**FIGURE 4:**
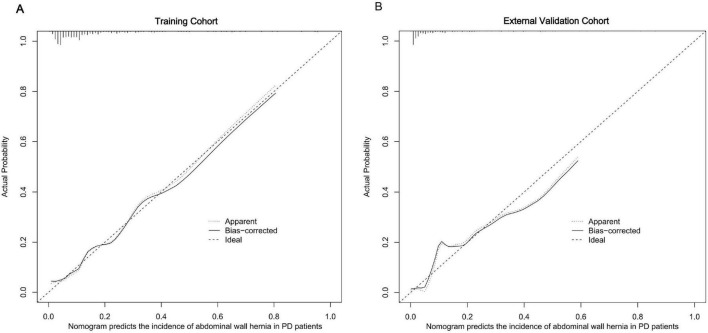
Calibration curves for predicting the incidence of abdominal wall hernias in PD patients in the training (A) and external validation cohorts (B).

**FIGURE 5:**
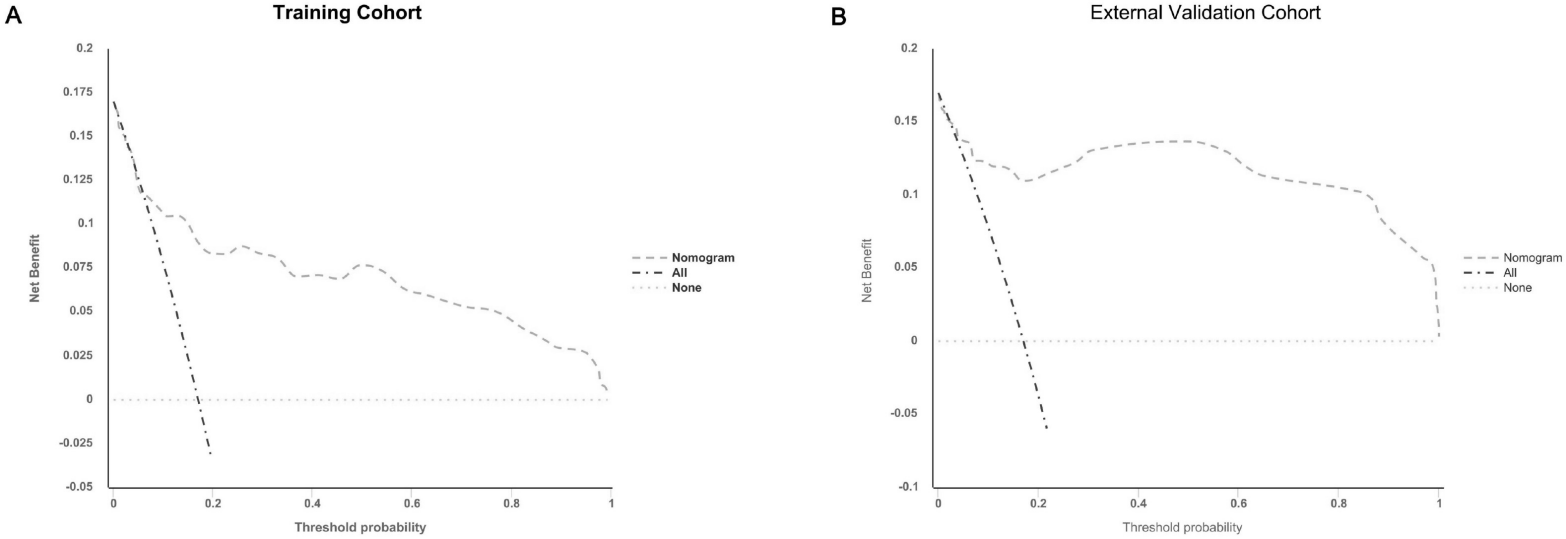
Decision curve analysis (DCA) plots for the nomogram in predicting the incidence of abdominal wall hernias in PD patients in the training (A) and external validation cohorts (B).

**FIGURE 6:**
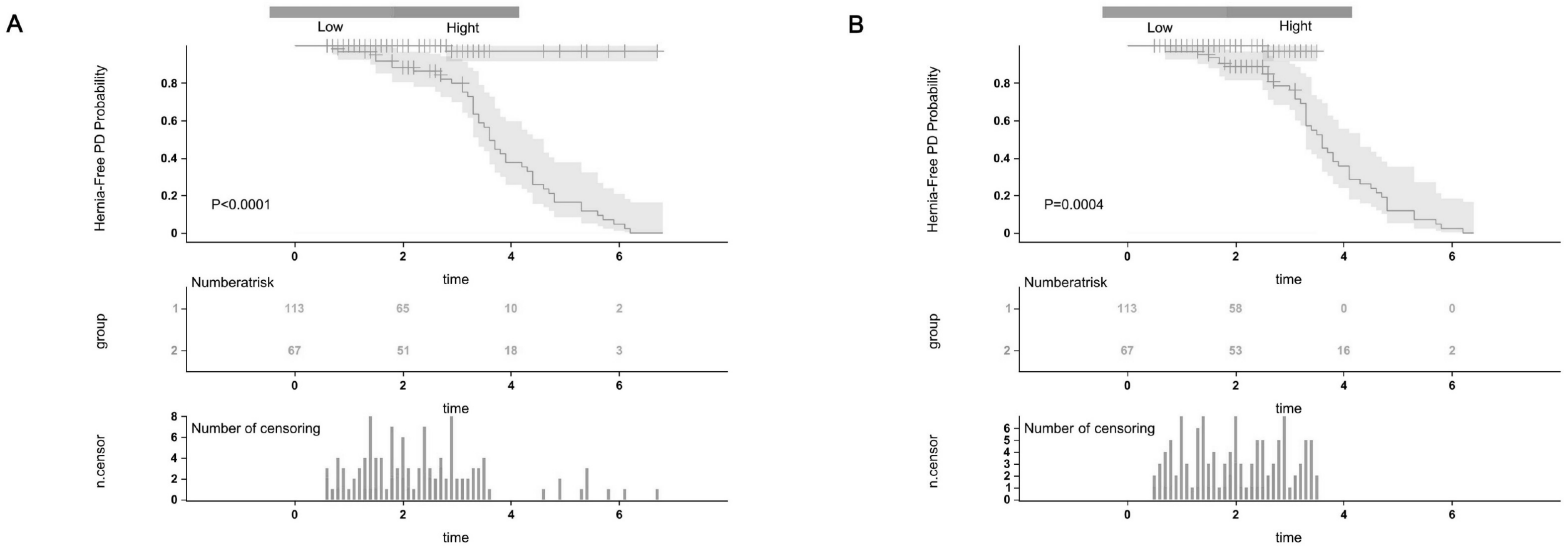
The Kaplan-Meier survival curves for low-risk and high-risk groups of patients with abdominal wall hernia on PD were based on our nomogram calculated risk scores. The Hernia-Free PD Probability Kaplan-Meier survival curves in the training cohort (A) and the external validation cohort (B).

The calibration plots further demonstrated excellent consistency between predicted probabilities and actual outcomes in both the training and validation cohorts (Figures 4A, B), reflecting robust calibration accuracy. Decision curve analysis supported the clinical usefulness of the nomogram by showing a higher net benefit across a broad range of threshold probabilities in both the training and external validation datasets (Figures 5A, B). To evaluate its risk stratification performance, we used X-tile software to classify patients into low- and high-risk groups according to their total nomogram scores. Kaplan-Meier survival analysis revealed statistically significant differences in hernia-free survival between the two risk groups. Patients in the low-risk group had the lowest incidence of hernia, whereas those in the high-risk group experienced the highest incidence, both in the training and validation cohorts (Figures 6A, B), thus validating the nomogram’s capacity for effective risk stratification.

Having comprehensively evaluated the predictive accuracy, calibration ability, clinical utility, and risk - stratification performance of the nomogram, it is now essential to delve deeper into the biological and clinical implications of these findings. The robust results obtained from the ROC curves, calibration plots, decision curve analysis, and Kaplan - Meier survival analysis set a solid foundation for understanding how this nomogram can be translated into real - world clinical practice.

In the subsequent discussion, we will explore the potential mechanisms that might explain the relationships between the variables in the nomogram and the development of abdominal wall hernias in peritoneal dialysis patients.

## 4 Discussion

Peritoneal dialysis is a vital therapeutic option for patients with end-stage renal disease. However, it is often complicated by the development of abdominal wall hernias, which not only increase hospitalization rates and surgical risks but can also compromise dialysis efficacy and long-term patient outcomes. Epidemiological studies indicate that the incidence of abdominal wall hernias among peritoneal dialysis patients ranges from 10% to 30%, frequently accompanied by high recurrence rates and significant economic burdens on healthcare systems. Despite the clinical significance of this complication, there remains a notable lack of robust, individualized risk prediction tools. The absence of such tools hinders the early identification of high-risk patients and the timely implementation of preventive measures, thereby limiting effective management.

To address this unmet clinical need, we developed the first clinically derived nomogram to predict the risk of abdominal wall hernia in patients undergoing peritoneal dialysis. The model demonstrated good discriminative performance, with AUCs of 0.823 and 0.747 in the training and external validation cohorts, respectively. However, the calibration curve (Figure 4) indicated a narrow range of predicted probabilities, suggesting limited variability in risk scores. This may stem from the relatively homogeneous distribution of key variables–such as age, dialysis modality, and serum albumin–within the study population, thereby constraining the model’s predictive spectrum.

The nomogram integrates multiple objective clinical variables, offering improved risk stratification over conventional single-variable approaches. Its consistent performance across independent validation cohorts supports its generalizability and potential as a clinical decision-support tool. These findings highlight the feasibility of individualized hernia risk prediction in peritoneal dialysis and lay the groundwork for future prospective validation.

Regarding the model’s development, we acknowledge that certain high-risk groups–such as those with prior abdominal surgery, connective tissue disorders, or major comorbidities–were excluded based on predefined inclusion and exclusion criteria. These criteria were established to minimize confounding effects from surgical history and to ensure a more homogeneous baseline population for model derivation. Nevertheless, the exclusion of these subgroups may limit the model’s applicability in more diverse or complex clinical scenarios. Future external validation in broader patient populations will be critical to assess the model’s robustness and generalizability across heterogeneous clinical settings.

An independent selection of risk factors was conducted using the logistic proportional hazards model, and the model’s performance was rigorously assessed through comprehensive metrics, including AUC, calibration curves, and decision curve analysis (DCA), thereby ensuring robust statistical validity. Moreover, external validation incorporating multicenter data further enhanced the model’s clinical applicability and generalizability. This study design represents a substantial improvement over previous investigations, which were often limited by small sample sizes and single-center data, thus providing a more solid evidence base to inform future clinical practice.

Existing studies have indicated that advancing age and hypoalbuminemia may represent potential risk factors for abdominal wall hernia (1, 2). Building upon this foundation, the present study further validates the weight of these factors within predictive models and incorporates PDV, establishing its role as an independent risk factor. From a physiological perspective, with increasing age, there is a decline in muscle mass and collagen synthesis capacity, leading to diminished tissue repair ability, compromised integrity of the abdominal wall structure, and consequently, an elevated risk of hernia development (8). Concurrently, an exacerbated chronic inflammatory state may impair tissue healing responses and promote the formation of weakened areas in the abdominal wall (9). On the other hand, hypoalbuminemia is frequently utilized as a biomarker for malnutrition and systemic inflammation; its reduction can suppress collagen synthesis, weaken the mechanical strength of connective tissues, and impair the wound healing process, thereby increasing the likelihood of abdominal wall hernia occurrence (10–12). Furthermore, chronic inflammation is prevalent among patients with end-stage renal disease, and through inducing muscle atrophy and protein catabolism, it exacerbates malnutrition and further compromises tissue repair capacity (13, 14). Maintaining optimal nutritional status, particularly by correcting hypoalbuminemia, may represent one important strategy in the prevention of abdominal wall hernias.

Notably, this study identifies continuous ambulatory peritoneal dialysis (CAPD) as a potential risk factor for the development of abdominal wall hernias when compared to automated peritoneal dialysis (APD). The observed association appears to stem from the intrinsic operational features of CAPD, particularly its characteristic prolonged dwell times and sustained intra-abdominal pressure (IAP) elevation. From a biomechanical standpoint, the persistent mechanical stress imposed during CAPD may induce chronic tissue strain that exceeds the adaptive capacity of the abdominal wall’s viscoelastic properties. This sustained pressure, compounded by repetitive manual exchanges that introduce fluctuating shear forces, may progressively compromise myofascial integrity and accelerate collagen matrix degradation (15, 16). Additionally, the absence of nocturnal decompression periods in CAPD may restrict opportunities for tissue recovery, thereby contributing to cumulative structural fatigue over time. These findings suggest that while CAPD may be associated with increased hernia risk, it likely acts in conjunction with other clinical and mechanical factors rather than as an independent predictor.

The repetitive pressurization of the peritoneal cavity inherent to CAPD may further impair connective tissue remodeling and hinder myofascial repair mechanisms (17). Increased dwell frequency, larger dialysate volumes, and continuous abdominal wall tension may interact synergistically to erode the mechanical stability of the anterior abdominal wall over the course of long-term dialysis. Although this study did not specifically assess peritoneal transport characteristics, previous evidence suggests that high transporter status-often managed with intensified exchange protocols-may amplify these biomechanical stresses, potentially potentiating tissue injury through a combination of chronic mechanical strain and inflammation-mediated extracellular matrix disruption (18). These complex interactions highlight the multifactorial nature of hernia development in peritoneal dialysis patients and caution against the interpretation of CAPD as an isolated causative factor.

Compared with previous studies, this study demonstrates notable advancements in both the integrity and clinical applicability of the model construction. Xu et al. (1) conducted a systematic review and meta-analysis, identifying hypoalbuminemia, advanced age, diabetes, and obesity as key risk factors for abdominal wall hernia in patients undergoing peritoneal dialysis; Chen et al. (2) further corroborated hypoalbuminemia and advanced age as independent predictors. Importantly, this study introduces peritoneal dialysis modality as a dynamic variable and develops a visually accessible nomogram for individualized risk assessment, thereby enhancing the feasibility and precision of clinical decision-making. Furthermore, this model allows for the incorporation of potential interventions, such as nutritional support aimed at correcting hypoalbuminemia-a modifiable risk factor that may significantly influence hernia development. Targeted dietary interventions or supplementation strategies could potentially mitigate the risk of herniation by improving serum albumin levels and enhancing abdominal wall integrity.

This work not only addresses a gap in the existing literature but also offers a practical tool to guide preventive and therapeutic strategies in clinical practice. In terms of clinical application, the nomogram developed in this study can be used for early risk screening in outpatient or inpatient settings, helping to identify high-risk patients and formulate targeted intervention strategies, such as enhancing nutritional support, optimizing dialysis regimens, avoiding excessive intra-abdominal pressure, and implementing prophylactic surgical reinforcement when necessary. This proactive management strategy holds promise for reducing the incidence of abdominal wall hernias and associated complications, thereby improving patients’ quality of life and prognosis (19–21).

Certainly, this study has several limitations that warrant discussion. First, the retrospective observational design inherently limits the ability to establish causal relationships, a common challenge in studies of this nature (22). Second, although our cohort included patients from multiple centers, the sample size remains relatively modest compared to large-scale epidemiological studies, and the inclusion criteria may introduce potential selection bias (23). Furthermore, several potentially important confounding factors, such as physical activity level, smoking history, and genetic predisposition, were not systematically collected or analyzed, which could influence hernia development and thus affect the generalizability of the model. In addition, key clinical parameters related to peritoneal function, such as peritoneal transport type, were not integrated into the current framework, limiting deeper mechanistic insights into the impact of dialysis modality on abdominal wall integrity.

To address these gaps, future research should incorporate larger, prospective multicenter cohorts with standardized data collection protocols, dynamic clinical monitoring, advanced imaging modalities, and novel biomarkers reflective of connective tissue metabolism and systemic inflammation. Such enhancements would not only improve model robustness but also support broader external validation efforts. Ultimately, the integration of radiomic features, machine learning techniques, and multi-omics data may offer a more comprehensive risk stratification tool tailored for clinical decision-making.

Looking ahead, it is recommended to explore an intelligent prediction system integrating radiomics, machine learning algorithms, and multi-omics information to enhance the accuracy and generalization ability of the model. Moreover, other research teams are encouraged to conduct external validation in diverse populations and design randomized controlled trials (RCTs) based on the risk stratification of the present model to evaluate its practical effectiveness in preventive interventions. This will provide a solid foundation for achieving precision medicine and individualized intervention.

In conclusion, the nomogram model established in this study offers a new tool for early warning of abdominal wall hernia in patients undergoing peritoneal dialysis, demonstrating promising clinical application potential and warranting broader implementation and further validation.

## Data Availability

The original contributions presented in this study are included in this article/Supplementary material, further inquiries can be directed to the corresponding author.
