# Supplementary material for: A study on predictive nomogram for abdominal wall hernia in peritoneal dialysis patients based on multicenter data
Source: Front Med (Lausanne). 2025 Dec 9;12:1624861. doi: 10.3389/fmed.2025.1624861 (PMC12722950; doi:10.3389/fmed.2025.1624861)
Supplement: Supplementary file 1 [file Table_1.docx]

Supplementary Material

## Supplementary Figures


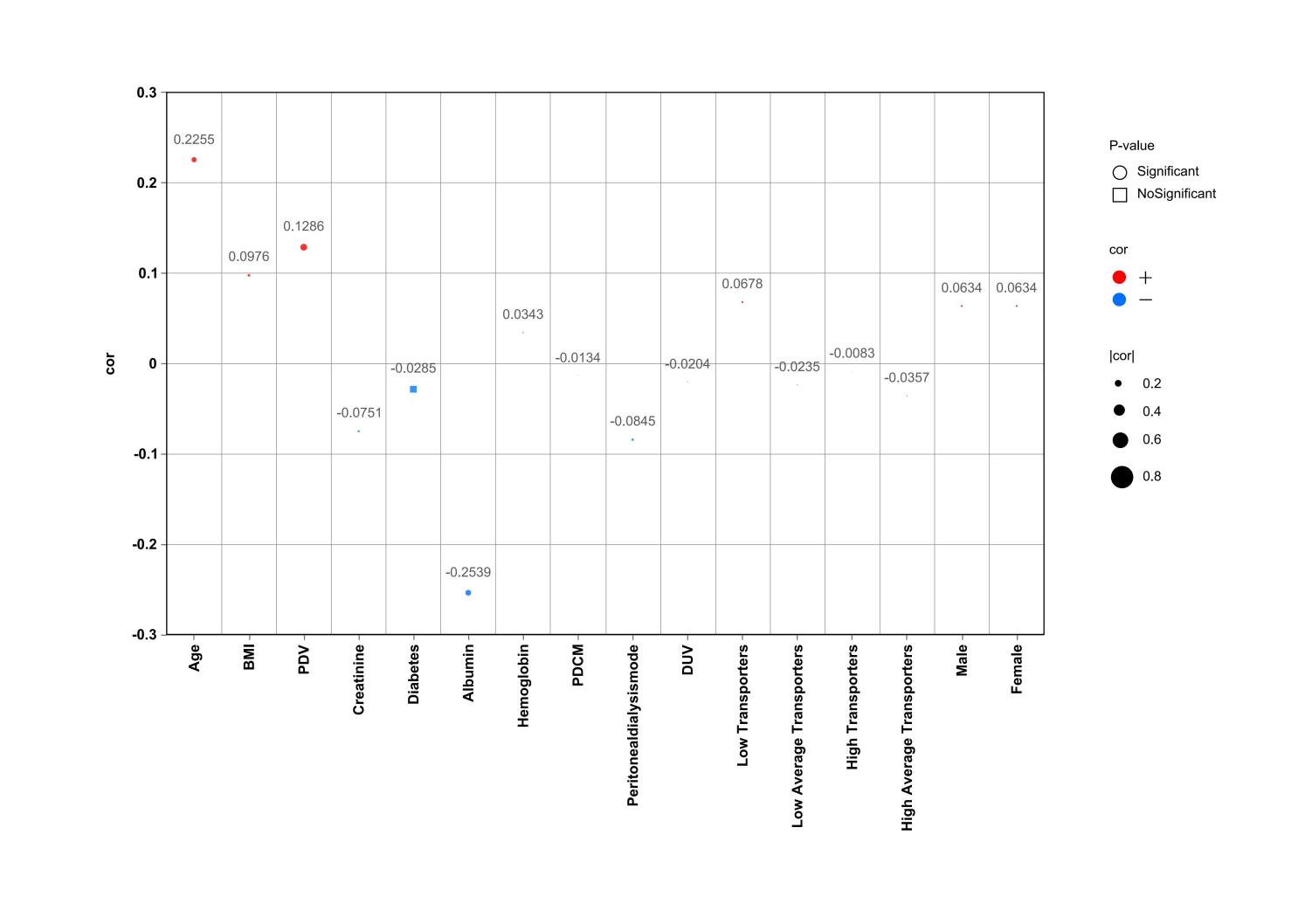


**Supplementary Figure 1.** Multivariate correlation bubble diagram of abdominal wall hernia related to peritoneal dialysis
